# The Spectrum of Co-Diagnoses in Patients with Colorectal Cancer: A Retrospective Cohort Study of 17,824 Outpatients in Germany

**DOI:** 10.3390/cancers14153825

**Published:** 2022-08-06

**Authors:** Sven H. Loosen, David Schöler, Simon Labuhn, Alexander Mertens, Markus S Jördens, Mark Luedde, Karel Kostev, Tom Luedde, Christoph Roderburg

**Affiliations:** 1 Clinic for Gastroenterology, Hepatology and Infectious Diseases, Medical Faculty of Heinrich Heine University Düsseldorf, University Hospital Düsseldorf, Moorenstrasse 5, 40225 Düsseldorf, Germany; 2 KGP Bremerhaven, 27574 Bremerhaven, Germany; 3 Epidemiology, IQVIA, Main Airport Center, Unterschweinstiege 2–14, 60549 Frankfurt, Germany

**Keywords:** CRC, rectal carcinoma, biomarker, polyneuropathy, screening

## Abstract

**Simple Summary:**

We identified a panel of co-diagnosis occurring at higher or lower frequencies in CRC patients compared to matched controls without CRC. Such data might help optimizing CRC screening and improving the clinical management of CRC patients.

**Abstract:**

**Background:** The prognosis of colorectal cancer (CRC) patients is determined to a decisive extent by comorbidities. On the other hand, anti-cancer treatments for CRC are associated with relevant toxicities and may therefore cause additional comorbidities. **Methods:** This retrospective cohort study assessed the prevalence of various diseases in patients 12 months before and 12 months after an initial diagnosis of colorectal cancer (ICD-10: C18, C20) in 1274 general practices in Germany between January 2000 and December 2018. The study is based on the Disease Analyzer database (IQVIA), which contains drug prescriptions, diagnoses, and basic medical and demographic data. Patients with and without CRC were matched by sex, age, and index year. **Results:** We identified several diagnoses with a significantly higher prevalence among CRC patients 12 months prior to the index date compared to controls. These diagnoses included gastrointestinal hemorrhage, hemorrhoids, perianal venous thrombosis, and abdominal and pelvic pain, as well as functional intestinal disorders. In contrast, the prevalence of lipid metabolism disorder, depression, hypertension, coronary heart disease, or acute bronchitis was significantly lower in CRC cases. After diagnosis of CRC, we found a significantly higher prevalence of anemia, polyneuropathies, functional intestinal disorders, and chronic kidney disease among CRC patients compared to the control group, while the prevalence of acute upper respiratory infections of multiple and unspecified sites and acute bronchitis was significantly lower in CRC patients compared to non-CRC patients. **Conclusions:** In the present study, we identified a variety of diseases occurring at higher or lower frequencies in CRC patients compared to matched controls without CRC. This might help to select patients for early CRC screening and improve the clinical management of CRC patients.

## 1. Introduction

Colorectal cancer represents the second most common cause of cancer-related death worldwide [1,2]. In Germany, 62,230 new cases and 25,972 deaths were registered in 2018 [1]. Large epidemiological studies have shown that numerous facets of the “Western lifestyle” such as insufficient physical activity, excessive consumption of animal fats and red meat, and insufficient intake of dietary fiber are important risk factors predisposing to the development of colon cancer [1]. The prognosis for patients with colorectal cancer has improved significantly in recent years after decades of stagnation. This progress has been achieved on the one hand by new targeted systemic therapies and on the other hand by the increasing use of highly active multimodal treatment approaches in the therapeutic course of many patients [3]. However, for multimorbid patients who cannot receive such intensive therapies, or can only receive them to a very limited extent, the prognosis remains poor [3]. Cardiovascular and pulmonary disease play a particular role here, sharing numerous risk factors with colorectal cancer. A more detailed analysis of the spectrum of co-diagnoses of patients with colorectal carcinoma may therefore provide valuable data regarding the possibilities of prevention of the tumor disease itself but also regarding the prevention of diseases that prevent optimal treatment of the tumor. High-intensity multimodal treatment procedures, which are standard today for many patients with colorectal carcinoma, place—as mentioned above—high demands on the physical resilience of the patients. On the other hand, they are also associated with a whole spectrum of side effects that can lead to significant limitations in the quality of life. Examples include oxaliplatin-associated polyneuropathy or chronic gastrointestinal complaints as a consequence of extensive bowel resections [3]. In this context, a more detailed investigation of the spectrum of co-diagnoses of patients after diagnosis of colorectal cancer may help to individualize and further optimize the clinical management of such patients.

## 2. Materials and Methods

### 2.1. Database

This study was based on data from the Disease Analyzer database (IQVIA), which contains drug prescriptions, diagnoses, and basic medical and demographic data obtained directly and in anonymous format from computer systems used in the practices of general practitioners and specialists (Rathmann et al., 2018). The database covers approximately 3% of all outpatient practices in Germany. It has previously been shown that the panel of practices included in the Disease Analyzer database is representative of general and specialized practices in Germany [4]. Finally, this database has already been used in previous studies focusing on cancer [5,6,7].

### 2.2. Study Population

**Figure 1:**
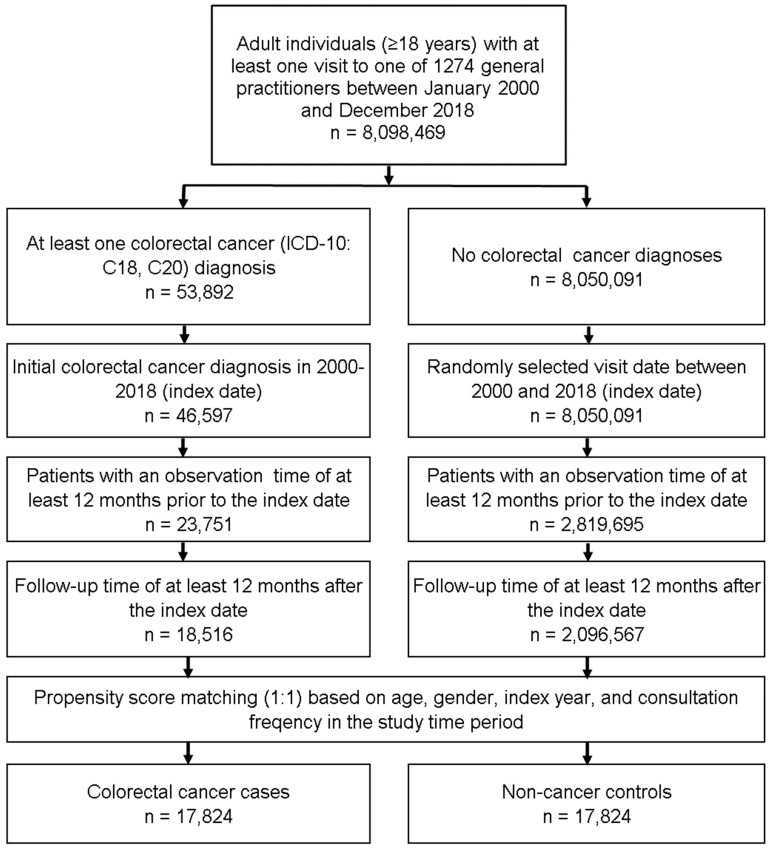
Selection of study patients.

This retrospective cohort study included adult individuals (≥18 years) with an initial diagnosis of colorectal cancer (CRC, ICD-10: C18, C20) in 1274 general practices in Germany between January 2000 and December 2018 (index date; Figure 1). Further inclusion criteria were an observation time of at least 12 months prior to and a follow-up time of at least 12 months after the index date. CRC patients were matched to individuals without CRC (controls) by propensity scores based on age, gender, index year, and consultation frequency within 12 months prior to and 12 months after the index date. For the control group, the index date was defined as a randomly selected visit between January 2000 and December 2018 (Figure 1).

### 2.3. Study Outcomes and Statistical Analyses

The outcomes of this study were various co-diagnoses documented within 12 months prior as well as 12 months after the index date (diagnosis of CRC) among CRC patients compared to non-CRC individuals. Differences with respect to the cohort characteristics between CRC patients and controls were tested using Chi-squared tests for categorical variables and Wilcoxon tests for continuous variables. All co-diagnoses with a prevalence of at least 3% in CRC patients or controls were included in the analysis. The index for potential differences between CRC and non-CRC individuals was defined as the prevalence in CRC patients divided by prevalence in controls. Differences of the prevalence of each diagnosis were tested using Chi-squared tests. To counteract the problem of multiple comparisons, *p*-values < 0.001 were considered statistically significant. Analyses were performed using SAS version 9.4 (SAS institute, Cary, NC, USA).

## 3. Results

### 3.1. Basic Characteristics of the Study Sample

The present analysis included a total of 17,824 patients with a documented diagnosis of colorectal cancer (CRC) as well as a propensity score matched cohort of 17,824 individuals without CRC. The basic characteristics of the study cohort are summarized in Table 1. 47.4% of patients were female. The mean age [SD] of the cohort was 69.3 [12.0] years; The average number of annual GP visits was 10 within 12 months prior to the index date (diagnosis of CRC) and 13 within 12 months after the index date.

### 3.2. Evaluation of Frequently Coded Co-Diagnoses within 12 Months Prior to Diagnosis of Colorectal Cancer

We first assess the prevalence of various co-diagnoses within 12 months prior to the index date. We identified several co-diagnoses with a significantly higher prevalence among CRC patients compared to controls. These co-diagnoses included gastrointestinal hemorrhage (4.7 vs. 0.6%), hemorrhoids and perianal venous thrombosis (3.7 vs. 1.5%), abdominal and pelvic pain (7.8 vs. 3.9%), other functional intestinal disorders (3.9 vs. 2.2%), gastroenteritis and colitis of infectious and unspecified origin (5.8 vs. 3.4%), and gastritis and duodenitis (7.6 vs. 6.5%, Table 2). In contrast, the prevalence of lipid metabolism disorder, depression, hypertension, coronary heart disease, acute bronchitis, or dorsalgia was significantly lower in CRC cases compared to controls (Table 2). The frequency of other co-diagnoses such as arterial fibrillation, COPD, or dizziness were comparable between CRC and non-CRC patients (Table 2).

### 3.3. Evaluation of Frequently Coded Co-Diagnoses within 12 Months after the Diagnosis of Colorectal Cancer

In a next step, we evaluated the prevalence of different co-diagnoses within 12 months following the index date (initial diagnosis of CRC). Here, we observed a significantly higher prevalence of iron deficiency anemia (6.8 vs. 2.2%), other anemias (4.0 vs. 2.1%), other and unspecified polyneuropathies (3.2 vs. 1.8%), diverticular disease of intestine (4.0 vs. 2.4%), other functional intestinal disorders (5.6 vs. 3.5%), chronic kidney disease (3.2 vs. 2.4%), and gastroenteritis and colitis of infectious and unspecified origin (5.8 vs. 4.8%) among CRC patients compared to the control group (Table 3). In contrast, the prevalence of acute upper respiratory infections of multiple and unspecified sites, acute bronchitis, dorsalgia, and osteoarthritis of the knee was significantly lower in CRC patients compared to non-CRC patients (Table 3). Again, we identified some other co-diagnoses such as arterial fibrillation, dizziness, or nontoxic goiter that were comparable between CRC and non-CRC patients (Table 3).

## 4. Discussion

In this study, we performed a comprehensive analysis on the prevalence of a broad panel of co-diagnoses in patients with colorectal cancer. Most importantly we show that gastrointestinal diseases including hemorrhage, abdominal and pelvic pain, and functional intestinal disorders were overrepresented in patients within 12 months before the diagnosis of CRC. Unexpectedly, frequencies of lipid metabolism disorder, depression, hypertension, and coronary heart disease were significantly lower in CRC cases compared to controls. Within 12 months after the diagnosis of CRC, more patients displayed anemia, polyneuropathies, and chronic kidney disease, most likely representing treatment-related complications in these patients. Such data might help to improve the clinical management of patients with CRC.

Gastrointestinal bleedings belong to the most important clinical symptoms occurring in the context of CRC [3]. Likewise, in our analyses, gastrointestinal hemorrhage was coded at significantly higher rates in patients with CRC when compared to controls. Interestingly, the diagnoses “hemorrhoids” and “perianal venous thrombosis” were also significantly overrepresented in the 12 months period before the index date in CRC patients. Similarly, rather unspecific gastrointestinal diseases including abdominal and pelvic pain, functional intestinal disorders (incl. constipation, diarrhea), gastroenteritis, colitis and duodenitis were found at higher frequencies in this group. These results suggest that at least in some patients, complaints caused by a CRC were initially attributed to other gastrointestinal diseases before the final diagnosis of CRC was made. Thus, our data support the role of early colonoscopy in patients with gastrointestinal symptoms and specifically in patients with gastrointestinal bleeding to ensure early CRC as recently described [3,8,9].

In contrast to various digestive tract cancers, the prognosis of CRC patients has constantly improved over the last years [10]. Presently, many different multimodal therapeutic approaches are available even in patients with metastasized disease stages, particularly in case of liver-limited disease [10,11,12]. Most importantly, even extensive tumor resection of liver metastases has not only proven efficacy to reduce the tumor burden, but may even be done with curative intent, potentially providing long-term survival [13,14]. Moreover, novel systemic treatment options were recently introduced into clinical algorithms for CRC patients, including specific antibodies or tyrosine kinase inhibitors, offering several lines of chemotherapy which can also lead to a considerably prolonged survival [15,16,17]. With all these options available, the individual patients’ characteristics such as age or comorbidities increasingly gain attention since they determine the patients’ ability to receive treatment. Here we demonstrate that—unexpectedly—patients with CRC display significantly lower frequencies of lipid metabolism disorders, arterial hypertension, and coronary heart disease all representing diagnoses potentially preventing the patient from receiving aggressive treatment. Nevertheless, it is important to note that the overall prevalence of this diseases in the CRC group is still high (e.g., hypertension with 31.4% and coronary heart disease 9%), clearly demonstrating that such co-diagnoses need to be considered in patients with CRC.

Oxaliplatin represent a cornerstone in the systemic treatment of patients with CRC, both in metastasized and non-metastasized disease stages [18]. Despite having proven efficacy in combination with 5-FU and/or different antibodies, the neurotoxic effect of oxaliplatin limits its use in many patients [19]. In line with these previous data, rates of polyneuropathies (ICD G62) were significantly higher in patients with CRC than in other patients. These data clearly underscore the important role of this toxicity in the context of CRC treatment and simultaneously prove the reliability of our data. Interestingly, next to polyneuropathies, chronic kidney disease was overrepresented in patients after diagnosis of CRC. It seems likely that this represents a direct consequence of anti-cancer treatments including, e.g., nephrotoxic substances or kidney malperfusion during or after surgery. Finally, functional intestinal disorders (obstipation, diarrhea) were coded at higher frequencies in CRC patients than in non-CRC patients after the index date. Of course, they might be related to postoperative gastrointestinal problems but also to psychological stress occurring after a cancer diagnosis.

We acknowledge important limitations of our study. First, our analysis is fully descriptive and does not allow to draw any conclusions on whether a certain disease might represent a risk factor for the development of CRC or was really caused by the presence of CRC or associated anti-cancer treatments. Moreover, database analyses as performed here are generally limited by a lack of completeness of the data on which they are based. For example, we cannot provide information on the individual disease stages or on the guideline-based therapy of the diseases and the course of the disease. As such, we are unable to differentiate between metastasized or non-metastasized CRC. Furthermore, no data were available in the database on the individual tumor localization, which might be of importance since left-sided CRC and right-sided CRC are regarded as fully different diseases in terms of tumor biology, response to treatment, or prognosis. In addition, identification of patients was based on officially coded diagnoses only, most likely resulting in a recording bias. The possibility that diagnoses have been misclassified within the ICD-10 coding system cannot be excluded. Finally, the depth of the analyses is partially limited because of the data source used, and we are therefore unable to comment on, for example, more precise locations of GI bleeding. However, we used data from a large database for our study. We are therefore confident that the data shown are reliable and clinically meaningful.

In summary, we provide a comprehensive and broad picture of the spectrum of co-diagnoses in patients before and after diagnosis of CRC. Co-diagnoses might both represent risk factors for the occurrence of CRC as well as aggravating factors for the course of the disease. Finally, they offer new starting points for multimodal treatment approaches for CRC in the future.

## Figures and Tables

**Table 1:** Basic characteristics of the study sample (after 1:1 propensity score matching).

Variable	Proportion among Individuals with Colorectal Cancer (%), *n* = 17,824	Proportion among Individuals without Colorectal Cancer (%), *n* = 17,824	*p*-Value
Age (Mean, SD)	69.3 (12.0)	69.2 (12.1)	0.479
Age ≤ 50	7.0	7.4	0.574
Age 51–60	15.0	15.2	
Age 61–70	25.8	25.9	
Age >70	52.2	51.5	
Women	47.4	47.4	0.975
Men	52.6	52.6	
Consultation frequency within 12 months prior to the index date (Mean, SD)	9.6 (8.8)	9.7 (8.8)	0.336
Consultation frequency within 12 months after the the index date (Mean, SD)	13.3 (10.4)	13.2 (10.4)	0.275

Proportions of patients given in %, unless otherwise indicated. SD: standard deviation.

**Table 2:** Prevalence of different co-diagnoses documented within 12 months before the diagnosis of colorectal cancer.

Diagnosis	Proportion among Individuals with Colorectal Cancer (%), *n* = 17,824	Proportion among Individuals without Colorectal Cancer (%), *n* = 17,824	Index	*p*-Value
**Diagnoses with significantly higher prevalence among CRC patients**				
Gastrointestinal hemorrhage (K92.2)	4.7	0.6	7.5	<0.001
Hemorrhoids and perianal venous thrombosis (K64)	3.7	1.5	2.5	<0.001
Abdominal and pelvic pain (R10)	7.8	3.9	2.0	<0.001
Other functional intestinal disorders (incl. constipation, diarrhea) (K59)	3.9	2.2	1.8	<0.001
Gastroenteritis and colitis of infectious and unspecified origin (A09)	5.8	3.4	1.7	<0.001
Gastritis and duodenitis (K29)	7.6	6.5	1.2	<0.001
**Diagnoses with significantly lower prevalence among CRC patients**				
Lipid metabolism disorder (E78)	14.0	15.6	0.9	<0.001
Depression (F32, F33)	5.9	6.7	0.9	<0.001
Hypertension (I10)	31.4	34.4	0.9	<0.001
Coronary heart disease (I25)	9.0	10.0	0.9	<0.001
Acute bronchitis (J20)	5.6	6.5	0.9	<0.001
Dorsalgia (M54)	13.9	15.7	0.9	<0.001
**Diagnoses without significantly altered prevalence among CRC patients**				
Nontoxic goiter (E04)	5.1	4.5	1.1	0.015
Diabetes mellitus (E10-E14)	13.9	14.0	1.0	0.714
Purine metabolism disorder (E79)	4.8	5.3	0.9	0.030
Sleep disorders (G47)	4.6	4.8	1.0	0.395
Atrial fibrillation and flutter (I48)	4.7	5.0	0.9	0.258
Heart failure (I50)	4.9	5.3	0.9	0.061
COPD (J44)	4.4	4.4	1.0	0.897
Gastro-esophageal reflux disease (K21)	6.1	6.2	1.0	0.709
Osteoarthritis of knee (M17)	3.9	4.5	0.9	0.005
Other disorders of urinary system (N39)	4.7	4.5	1.0	0.448
Dizziness (R42)	3.2	3.5	0.9	0.125

**Table 3:** Prevalence of different co-diagnoses documented within 12 months after the diagnosis of colorectal cancer.

Diagnosis	Proportion among Individuals with Colorectal Cancer (%), *n* = 17,824	Proportion among Individuals without Colorectal Cancer (%), *n* = 17,824	Index	*p*-Value
**Diagnoses with significantly higher prevalence among CRC patients**				
Iron deficiency anemia (D50)	6.8	2.2	3.1	<0.001
Other anemias (D64)	4.0	2.1	1.9	<0.001
Other and unspecified polyneuropathies (G62)	3.2	1.8	1.8	<0.001
Diverticular disease of intestine (K57)	4.0	2.4	1.7	<0.001
Other functional intestinal disorders (incl. constipation, diarrhea) (K59)	5.6	3.5	1.6	<0.001
Chroic kidney disease (N18)	3.2	2.4	1.4	<0.001
Gastroenteritis and colitis of infectious and unspecified origin (A09)	5.8	4.8	1.2	<0.001
**Diagnoses with significantly lower prevalence among CRC patients**				
Acute upper respiratory infections of multiple and unspecified sites (J06)	4.4	7.0	0.6	<0.001
Acute bronchitis (J20)	5.4	8.3	0.6	<0.001
Dorsalgia (M54)	14.7	20.8	0.7	<0.001
Osteoarthritis of knee (M17)	4.6	6.2	0.7	<0.001
Bronchitis, not specified as acute or chronic (J40)	3.3	4.3	0.8	<0.001
Lipid metabolism disorder (E78)	15.4	19.2	0.8	<0.001
Purine metabolism disorder (E79)	5.5	6.6	0.8	<0.001
COPD (J44)	4.8	5.8	0.8	<0.001
Hypertension (I10)	35.3	40.7	0.9	<0.001
Coronary heart disease (I25)	10.9	12.8	0.9	<0.001
Diabetes mellitus (E10-E14)	15.7	17.3	0.9	<0.001
**Diagnoses without significantly altered prevalence among CRC patients**				
Abdominal and pelvic pain (R10)	5.6	5.3	1.1	0.218
Gastritis and duodenitis (K29)	9.5	9.1	1.0	0.266
Nontoxic goiter (E04)	5.5	5.6	1.0	0.611
Gastro-esophageal reflux disease (K21)	8.4	8.1	1.0	0.441
Other disorders of urinary system (N39)	6.7	6.6	1.0	0.332
Dizziness (R42)				
Depression (F32, F33)	8.8	9.2	1.0	0.189
Somatoform disorders (F45)	4.1	4.6	0.9	0.024
Sleep disorders (G47)	6.2	7.0	0.9	0.003
Atrial fibrillation and flutter (I48)	6.5	7.0	0.9	0.076
Heart failure (I50)	6.6	7.0	0.9	0.070

## Data Availability

The datasets used and analyzed during the current study are available from the corresponding author on reasonable request.
